# Chest CT texture-based radiomics analysis in differentiating COVID-19 from other interstitial pneumonia

**DOI:** 10.1007/s11547-021-01402-3

**Published:** 2021-08-04

**Authors:** Damiano Caruso, Francesco Pucciarelli, Marta Zerunian, Balaji Ganeshan, Domenico De Santis, Michela Polici, Carlotta Rucci, Tiziano Polidori, Gisella Guido, Benedetta Bracci, Antonella Benvenga, Luca Barbato, Andrea Laghi

**Affiliations:** 1 Department of Medical-Surgical Sciences and Translational Medicine, Sapienza University of Rome - Sant’Andrea University Hospital, Via di Grottarossa, 1035-1039, 00189 Rome, Italy; 2 Institute of Nuclear Medicine, University College London Hospitals NHS Trust, London, UK

**Keywords:** COVID-19, Texture analysis, Diagnostic tool, Computed tomography

## Abstract

**Purpose:**

To evaluate the potential role of texture-based radiomics analysis in differentiating Coronavirus Disease-19 (COVID-19) pneumonia from pneumonia of other etiology on Chest CT.

**Materials and methods:**

One hundred and twenty consecutive patients admitted to Emergency Department, from March 8, 2020, to April 25, 2020, with suspicious of COVID-19 that underwent Chest CT, were retrospectively analyzed. All patients presented CT findings indicative for interstitial pneumonia. Sixty patients with positive COVID-19 real-time reverse transcription polymerase chain reaction (RT-PCR) and 60 patients with negative COVID-19 RT-PCR were enrolled.

CT texture analysis (CTTA) was manually performed using dedicated software by two radiologists in consensus and textural features on filtered and unfiltered images were extracted as follows: mean intensity, standard deviation (SD), entropy, mean of positive pixels (MPP), skewness, and kurtosis. Nonparametric Mann–Whitney test assessed CTTA ability to differentiate positive from negative COVID-19 patients. Diagnostic criteria were obtained from receiver operating characteristic (ROC) curves.

**Results:**

Unfiltered CTTA showed lower values of mean intensity, MPP, and kurtosis in COVID-19 positive patients compared to negative patients (*p* = 0.041, 0.004, and 0.002, respectively). On filtered images, fine and medium texture scales were significant differentiators; fine texture scale being most significant where COVID-19 positive patients had lower SD (*p* = 0.004) and MPP (*p* = 0.004) compared to COVID-19 negative patients. A combination of the significant texture features could identify the patients with positive COVID-19 from negative COVID-19 with a sensitivity of 60% and specificity of 80% (*p* = 0.001).

**Conclusions:**

Preliminary evaluation suggests potential role of CTTA in distinguishing COVID-19 pneumonia from other interstitial pneumonia on Chest CT.

## Introduction

A novel coronavirus, Severe Acute Respiratory Syndrome Coronavirus 2 (SARS-CoV-2) [1], was identified as the causative agent of several clinical conditions, the most common of which is pneumonia, reported as Coronavirus Disease-19 (COVID-19) [2, 3]. On January 2020, WHO declared the Chinese outbreak of COVID-19 to be a public health emergency of international concern.

Factors that could stem COVID-19 spread are: early detection, patients’ isolation, prompt treatment, and implementation of a robust system to trace contacts [4]. The reference standard for the diagnosis of SARS-CoV-2 infection is next-generation sequencing or real-time reverse transcription polymerase chain reaction (RT-PCR) methods applied to respiratory tract specimens [5]. However, due to intrinsic limitations of the method (i.e., site of specimen swabs, collection and transportation of samples and diagnostic kit performance), sensitivity of RT-PCR ranges between 63 and 78% [6, 7].

As an adjunct diagnostic tool in suspicious patients, Chest CT has gained an important role in COVID-19 pandemic thanks to its high sensitivity (97%) in COVID-19 diagnosis [8, 9]; the most frequent CT findings are multiple subpleural ground-glass opacities (GGO) associated with crazy paving pattern, consolidations, and small pulmonary vessels enlargement [2]. However, infected patients may show normal CT scans, particularly in the early phase of the disease [10]; moreover, Chest CT showed low specificity (25–56%) [11] since other lung infective and non-infective diseases may show similar appearance on Chest CT qualitative assessment [12–14].

Quantitative CT, radiomics, and artificial intelligence software analyses can potentially be effective in assessing severity of disease, increasing CT specificity, and strengthening the role of Chest CT in COVID-19 [15–21]. Among them, CT texture analysis (CTTA) is an emerging area of radiomics that extracts, analyses, and interprets quantitative imaging features not perceivable to the naked human eye.

CTTA allows objective assessment of the distribution of pixel/voxel intensity within a region of interest (ROI), reflecting the underlying biologic heterogeneity related to tissue microenvironment [22–24]. Radiomics machine learning-based tool has been recently studied to evaluate the severity of COVID-19 [25]. A recent study proposed by Wu et al. [26] have shown that despite the similar clinical and radiological manifestations in COVID-19 and non-COVID-19 patients, CT texture features may be a helpful toll to differentiate these two population. As a noninvasive and rapid imaging biomarker, texture analysis could improve COVID-19 chest CT diagnosis reducing false positive with the similar lung imaging pattern and allowing a better management of the disease.

Thus, the aim of our pilot study is to evaluate the potential role of texture-based radiomics analysis in distinguishing COVID-19 interstitial pneumonia from pneumonia of other etiology on Chest CT.

## Materials and methods

### Study population

This study was approved by our local institutional review board (IRB) and written informed consent was waived due to the retrospective nature. From March 8, 2020, to April 25, 2020, individuals with respiratory symptoms (cough and dyspnea) and fever suspected for COVID-19 and admitted to Emergency Department of Sant'Andrea University Hospital of Rome were enrolled and underwent unenhanced Chest CT.

To assess SARS-CoV-2 infection, real-time reverse transcriptase RT-PCR (Charitè, Berlin, Germany) [27] was performed on nasopharyngeal and oropharyngeal swabs collected from individuals on arrival in the Emergency Department and repeated after 24 h. Patients were considered SARS-CoV-2 negative after two consecutive negative RT-PCR results.

**Fig. 1:**
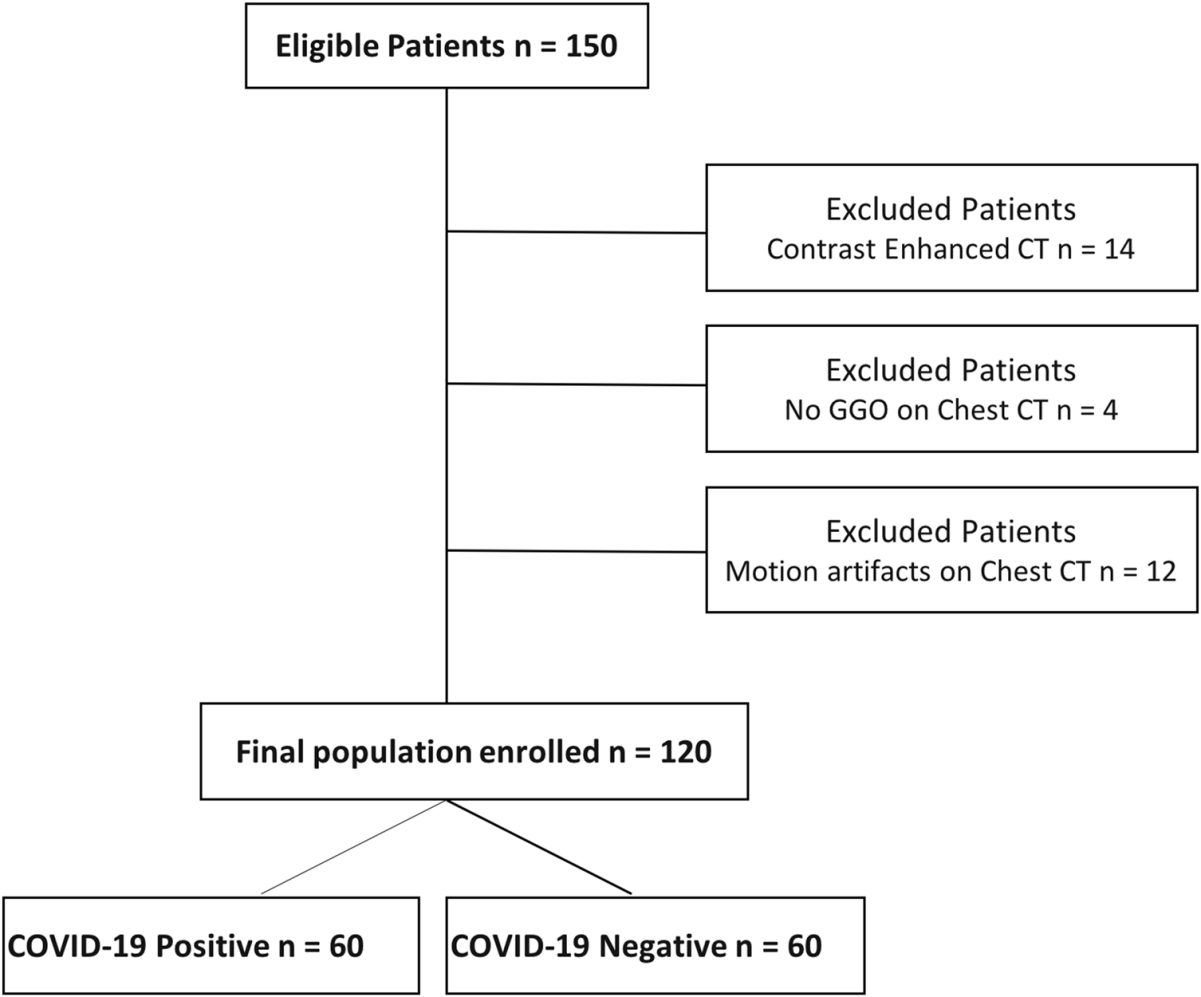
Patients enrollment flowchart

For the purpose of the study, inclusion criteria were: (a) availability of RT-PCR results, (b) unenhanced Chest CT performed on arrival, and c) the presence of GGO appreciable on Chest CT. Exclusion criteria were as follows: (a) Chest CT performed with contrast medium injection (e.g., pulmonary embolism suspicion), (b) motion artifacts on Chest CT and consequent poor image quality unsuitable for CTTA. The study population enrollment flowchart is summarized in Fig. 1.

Epidemiological and laboratory data of enrolled patients were collected.

### Chest CT protocol

All CT exams were performed with a 128-slice CT scanner (GE Revolution EVO 128 Slice CT Scanner, GE Medical Systems, Milwaukee, WI, USA). Datasets were acquired with patients placed in supine position in full inspiration, applying a standard volumetric protocol for unenhanced Chest CT, with fixed scan parameters (tube voltage: 120 kV, tube current modulation: 100–250 mAs, spiral pitch factor: 0.98, collimation width: 0.625).

Images were reconstructed applying the BONEPLUS convolution kernel, with a slice thickness of 1.25 mm and a spacing of 1.25 mm. Digital Imaging and Communications in Medicine (DICOM) image data were transferred into a picture archiving and communication system (PACS) workstation (Centricity Universal Viewer v.6.0, GE Medical Systems, Milwaukee, WI, USA).

### Texture analysis

**Fig. 2:**
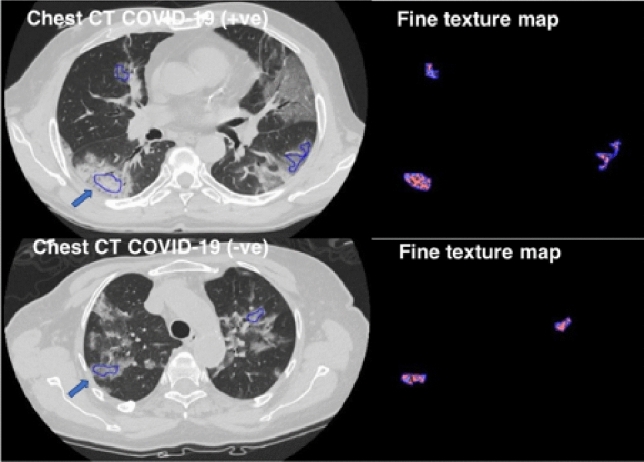
Workflow demonstrating the process of texture-based radiomics analysis on Chest CT in two patients—one diagnosed as COVID-19 positive (above) and other diagnosed as COVID-19 negative (below). ROIs (arrows) were manually contoured and kept approximately 2 mm within the margin of the GGO, to exclude from the analysis adjacent structures, such as vessel or bronchial branches, cavities, or normal lung parenchyma

DICOM images extracted from PACS were anonymized and transferred to a dedicated texture analysis research software (TexRAD, Feedback Medical Ltd., Cambridge, UK). Two expert radiologists (F.P. and D.C., with 5 and 10 years of experience in thoracic imaging, respectively) performed CTTA on the ROIs, in consensus reading, by encompassing the GGO displayed on the axial Chest CT images with fixed lung window (width: 1500 HU; level: − 600 HU). ROIs were manually contoured and kept approximately 2 mm within the margin of the GGO, to exclude from the analysis adjacent structures, such as vessel or bronchial branches, cavities, or normal lung parenchyma (Fig. 2). For each patient, five ROIs were drawn, one for each lung lobe. If GGOs were absent in a lung lobe, remaining ROIs were placed in the most affected lung lobe.

Heterogeneity within each ROI was evaluated using a filtration-histogram-based textural analysis technique. Filtration step using a band-pass Laplacian of Gaussian (LoG) filter comprised of extracting and enhancing image features of different sizes and intensity variation, corresponding to the spatial scale of the filter (SSF in radius). The feature scales ranged from SSF = 2 to 4 mm, where fine texture scale corresponded to SSF = 2 mm, medium texture scale corresponded to SSF = 3 mm, and coarse texture scale corresponded to SSF = 4 mm. Following the filtration step, texture quantification was computed using statistical- and histogram-based parameters at each derived image, as well as on the conventional unfiltered image (SSF = 0).

Statistical and histogram parameters comprised mean intensity, standard deviation (SD), entropy, mean of positive pixels (MPP), kurtosis, and skewness [23]. Figure 2 shows the filtration-histogram-based CTTA of COVID-19 positive and a COVID-19 negative patient.

### Statistical analysis

Patient demographics and clinical parameters were presented as follows: categorical variables as numbers and percentage whereas continuous variables as average and standard deviation. Nonparametric Mann–Whitney test assessed the ability of CTTA parameters and CT density (Hounsfield Unit, HU) to differentiate between the patient groups (positive COVID-19 versus negative COVID-19). Box and Whisker plots visualized the difference and trend between the two patient groups for each significant parameter. Diagnostic criteria for a composite score developed by combining the most significant parameters (using the median-value for each parameter from the entire patient population as a cutoff to categorize patients as positive COVID-19 and negative COVID-19) were established using a receiver operating characteristic (ROC) analysis which computed the area under the curve (AUC), cutoff (number of significant predictors), sensitivity, specificity, *p* value. Statistical analysis was carried out using SPSS (IBM Corp. Released 2017. IBM SPSS Statistics for Macintosh, Version 25.0. Armonk, NY: IBM Corp.) with a *p* < 0.05 considered to be significant.

## Results

### Study population

Initial study population comprised 150 patients. However, according to the exclusion criteria, 14 patients were excluded due to a contrast enhanced CT, 4 patients had no lung GGOs, and 12 patients were excluded due to motion artifacts on Chest CT, as shown in the flowchart below (Fig. 1). Therefore, final population consisted of 120 patients with GGO on Chest CT, of which 60 were COVID-19 positive and 60 were COVID-19 negative.

The positive group included 32 males and 28 females with a mean age of 65 ± 15 years, while the negative group listed 40 males and 20 females with a mean age of 70 ± 19 years, as shown in Table 1.

**Table 1:** Clinical parameters and blood test results of positive COVID-19 and negative COVID-19 patients

	COVID-19 positive		COVID-19 negative	
*Patients demographics*	*N. patients*	*%*	*N. patients*	*%*
Mean age	65 ± 15 y		70 ± 19 y	
Years (range)	23–94		18–98	
Number patients	60	100	60	100
Male	32/60	53	40/60	66
Female	28/60	47	20/60	34
*Blood test*				
*C-reactive protein (mg/L; normal range 0.00–0.50)*				
Increased	58/60	97	54/60	90
Normal	2/60	3	6/60	10
*Lactic acid dehydrogenase (U/L; range 125–220)*				
Increased	56/60	93	49/60	82
Normal	4/60	7	11/60	18
*Lymphocytes (*× *10*^*3*^*/mm*^*3*^*, normal range 1.5–3.0)*				
Increased	1/60	1	2/60	3
Decreased	49/60	82	40/60	67
Normal	10/60	17	18/60	30
*D-dimer (ng/ml, normal* < *243)*				
Increased	40/60	67	48/60	80
Normal	20/60	33	12/60	20
*Symptoms*				
Fever (> 37.5°)	15/60	25	11/60	18
Cough	32/60	53	36/60	60
Dyspnea	41/60	68	31/60	52

Among positive COVID-19 patients 41 (68%) presented dyspnea, 32 (53%) had cough, and 15 (25%) showed fever (> 37.5 °C). Laboratory blood tests showed typical lymphocytopenia in 49 (82%) patients, high C-reactive protein (> 0.50 mg/dL) in 58 (97%), rise of lactate dehydrogenase (> 220 U/L), and D-dimer (> 243 ng/ml) in 56 (93%) and 40 (67%) patients, respectively.

Among negative COVID-19 patients 36 (60%) had cough, 31 (52%) presented dyspnea, and 11 (18%) had fever. C-reactive protein, lactate dehydrogenase and D-dimer were above the threshold values in 54 (90%), 49 (82%), and 48 (80%) patients, respectively. Lymphocytopenia was found in 40 (67%) patients. The complete results are listed in Table 1.

### Image analysis

**Fig. 3:**
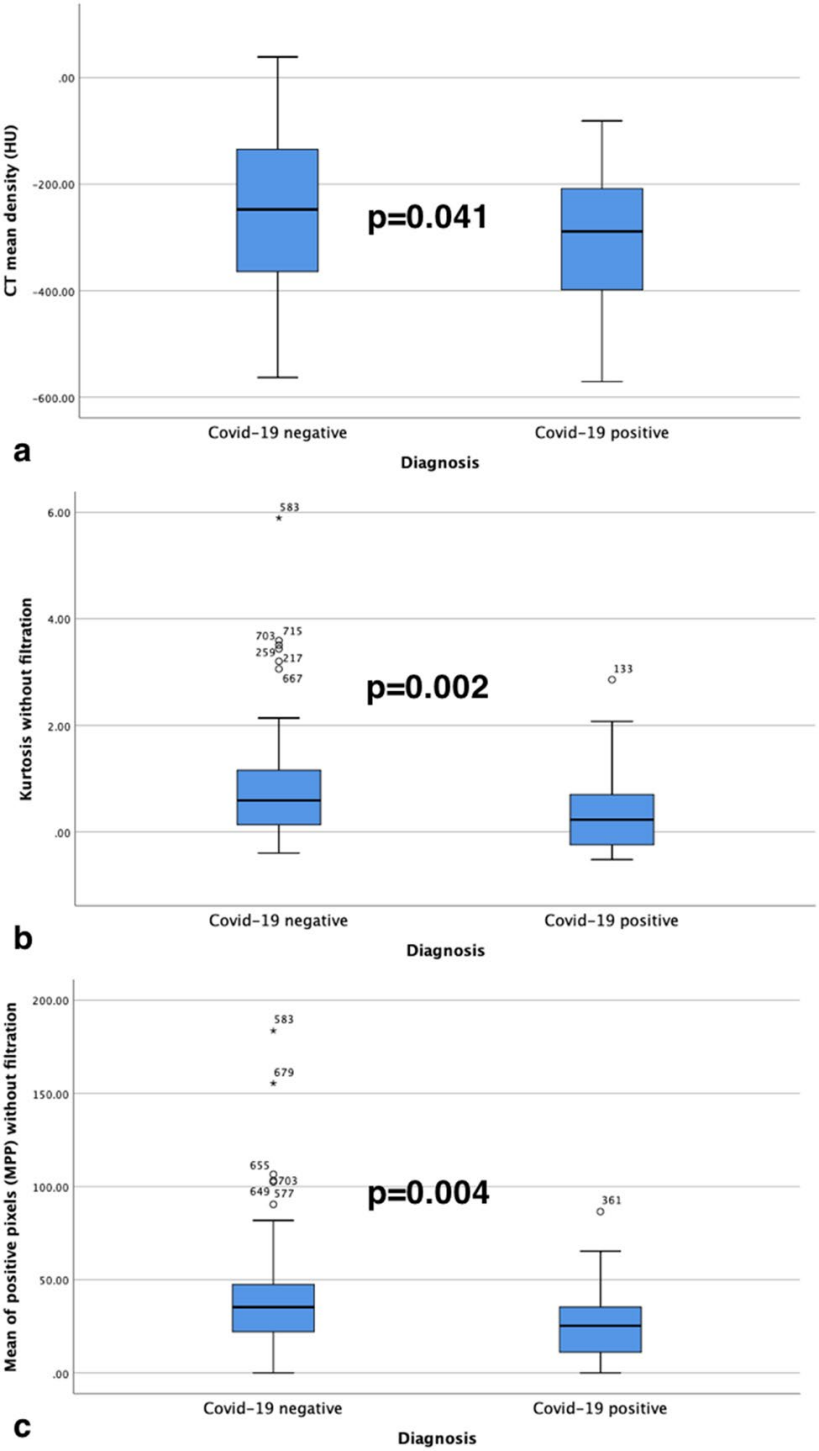
Box and Whisker plots highlight the significant difference between *Mean CT density* (**a**), texture parameters *Kurtosis* (**b**) and *Mean of Positive Pixels* (MPP) (**c**) without filtration for COVID-19 diagnosis

On unfiltered images, COVID-19 positive patients had a lower mean intensity (− 289 HU vs. − 247 HU; *p* = 0.041), MPP (25.3 vs. 35.3; *p* = 0.004), and kurtosis (0.2 vs. 0.6; *p* = 0.002) compared to negative COVID-19 individuals (Fig. 3).

**Fig. 4:**
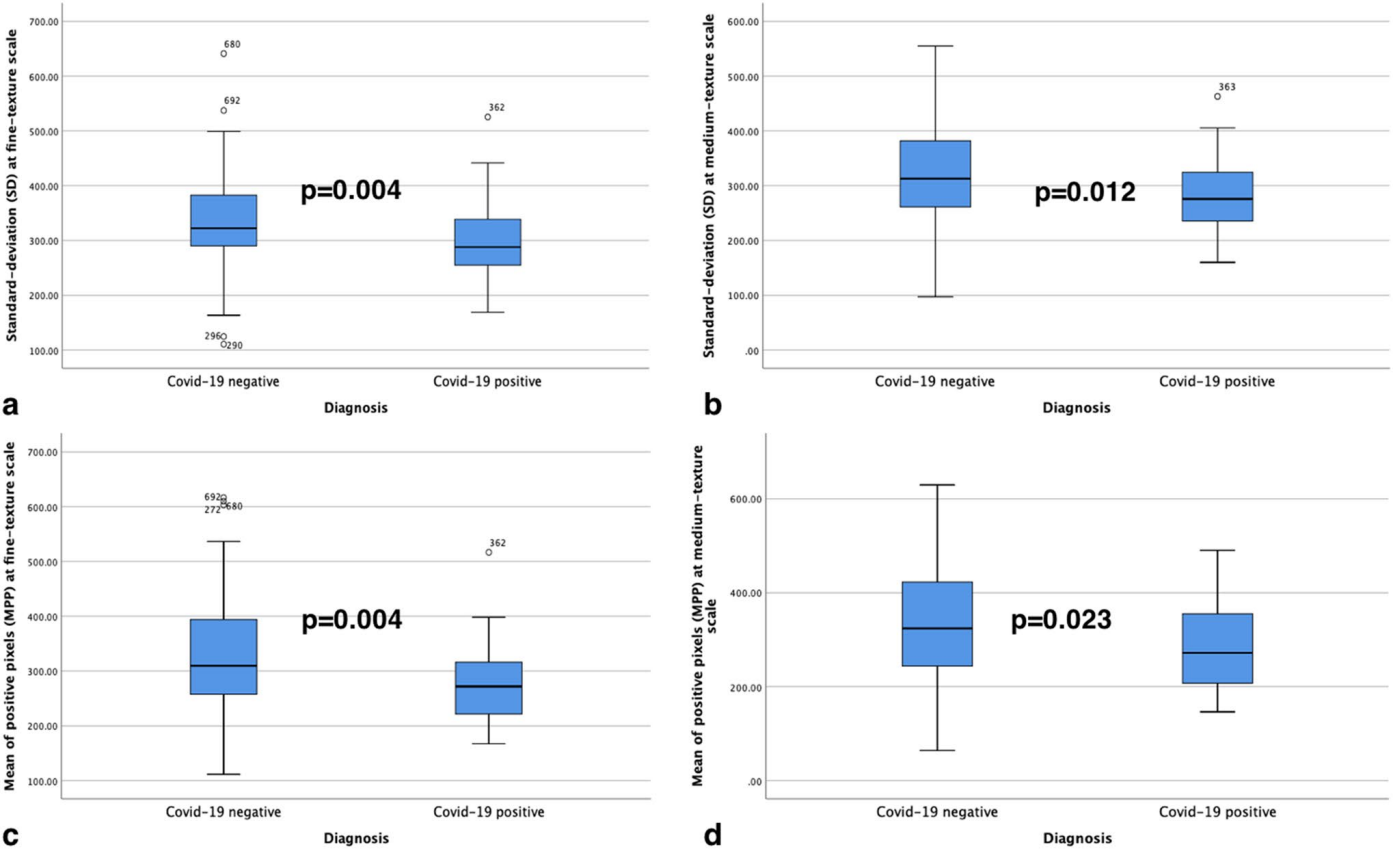
Box and Whisker plots highlight the significant differences at fine and medium texture parameters in terms of *Standard Deviation* (SD) (**a** and **b**) and *Mean of Positive Pixels* (*MPP*) (**c** and **d**) for COVID-19 diagnosis

On fine texture scale, COVID-19 positive patients had a lower SD (287.9 vs. 322.2; *p* = 0.004) and MPP (271.7 vs. 309.5; *p* = 0.004). The comparable results have been obtained on medium texture scale, in which COVID-19 positive patients accounted for lower SD (275.7 vs. 312.6; *p* = 0.012) and MPP (272.0 vs. 324.2; *p* = 0.023) compared to negative COVID-19 patients (Fig. 4). Coarse texture scale provided no significant differences in texture parameters between the two groups. The comprehensive results are summarized in Table 2.

**Table 2:** Summary of results (median) for Chest CT texture parameters within the two patient diagnostic groups

CT texture parameters	COVID-19 positive (median)	COVID-19 negative (median)	*p* value
*Without filtration—SSF* = *0*			
Mean intensity (HU)	− 288.710	− 247.353	**0.041**
Standard deviation	111.783	125.147	0.052
Entropy	4.733	4.758	0.954
Mean of positive pixels	25.313	35.302	**0.004**
Skewness	− 0.059	− 0.055	0.324
Kurtosis	0.227	0.590	**0.002**
*Fine texture scale—SSF* = *2*			
Mean intensity	44.009	86.771	0.114
Standard deviation	287.950	322.172	**0.004**
Entropy	4.869	4.928	0.618
Mean of positive pixels	271.700	309.479	**0.004**
Skewness	0.027	0.075	0.713
Kurtosis	0.202	0.131	0.797
*Medium texture scale—SSF* = *3*			
Mean intensity	81.300	119.381	0.155
Standard deviation	275.755	312.614	**0.012**
Entropy	4.857	4.918	0.609
Mean of positive pixels	272.036	324.228	**0.023**
Skewness	− 0.045	− 0.050	0.834
Kurtosis	− 0.150	− 0.071	0.648
*Coarse texture scale—SSF* = *4*			
Mean intensity	69.778	123.457	0.174
Standard deviation	255.785	284.804	0.091
Entropy	4.834	4.934	0.513
Mean of positive pixels	258.064	306.420	0.108
Skewness	− 0.138	− 0.148	0.867
Kurtosis	− 0.289	− 0.285	0.871

Statistically significant results based on Mann–Whitney test are highlighted in bold

**Fig. 5:**
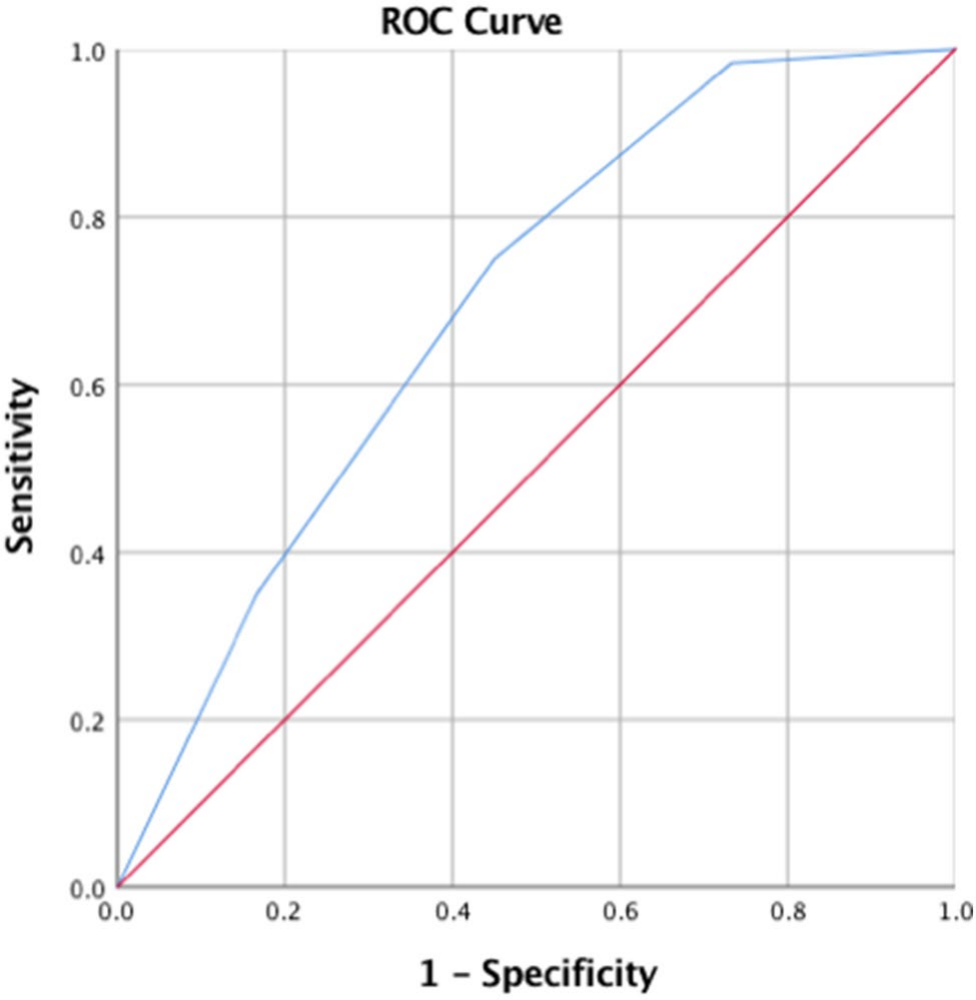
ROC analysis for the composite score was developed by combining the most significant texture parameters [fine texture: *Mean of Positive Pixels (MPP)* and *Standard Deviation*; without filtration texture: MPP and *Kurtosis*]. The presence of three or more risk factors identified patients with positive COVID-19 from patients with negative COVID-19 with a sensitivity of 60% and specificity of 80% (AUC = 0.7, *p* < 0.001)

A composite score developed by combining the most significant differentiators (fine texture scale: MPP < 290.122 and SD < 306.547; unfiltered images: MPP < 27.576 and kurtosis < 0.356) could differentiate COVID-19 positive patients from negative patients (*p* < 0.001). Specifically, the presence of any three or more of the above risk factors could identify COVID-19 positive patients from negative patients with a sensitivity of 60% and specificity of 80% (AUC = 0.7, *p* < 0.001; Fig. 5).

## Discussion

This pilot study showed the ability of CTTA to differentiate between COVID-19 positive and negative patients. SD and MPP at fine and medium filter scales had significantly lower values in positive COVID-19 patients compared to subject with interstitial pneumonia of other etiology, particularly at fine texture scale. Additionally, both kurtosis and MPP were lower in positive COVID-19 patients in the unfiltered image dataset. Such results, along with lower CT density in positive COVID-19 subjects, potentially reflects variation in GGO/parenchymal heterogeneity between the two groups. A combined score including the most significant texture parameters (presence of three or more risk factors) showed a good diagnostic performance in the identification of COVID-19 positive patients.

The last decade has seen a rapid growth of CTTA, especially applied in oncologic imaging, aiming to assess solid tumors’ heterogeneity and aggressiveness [28–34]. In thoracic oncology, CTTA has demonstrated the feasibility in predicting survival [35] and response to anti-angiogenic chemotherapy and immunotherapy [36–38] in lung cancer, in distinguishing lung cancer recurrence from post-radiation fibrosis [39], and the ability to assess pulmonary sub-nodules aggressiveness [40].

CTTA has been also applied in diffuse lung disease, such as pulmonary emphysema, pulmonary idiopathic fibrosis, and pulmonary embolism [41, 42]. Particularly, CTTA of pulmonary angiograms has been proved effective in providing correlates for ventilated and vascular lung, similar to a ventilation–perfusion V/Q scan, aiding in the diagnosis of pulmonary embolism in the presence of other causes of altered vascularity such as emphysema [42]. Other studies have explored the feasibility of CTTA to differentiate diffuse pulmonary alterations that appears very similar on visual assessment. Kloth et al. proved CTTA able to differentiate active alveolitis and lung fibrosis in patients with systemic sclerosis [43], as well alveolar hemorrhage from Pneumocystis jirovecii pneumonia [44], overcoming diagnostic challenges present on mere visual assessment due to overlapping imaging findings between the two entities at early stages. Therefore, CTTA might play a significant role in early differentiation of various pulmonary conditions manifesting with GGO, requiring different treatments and optimizes patient management.

Another investigation [45] applied CTTA in the identification of pulmonary abnormalities caused by H1N1 influenza on Chest CT, demonstrating that mean intensity, SD, and non-uniformity could differentiate between abnormal regions on H1N1 influenza patients from pulmonary fibrosis, normal and non-influenza infections. In accordance with previous investigation, in our study key differentiators were texture metrics (MPP, SD, and kurtosis) without and with filtration (fine and medium texture scales). These CTTA metrics reflect heterogeneity (intensity variation, irregularity, and tissue-contrast) in the distribution of parenchymal attenuation. Above studies demonstrate how CTTA extracts subtle image characteristic not perceivable to the naked eye and might aid radiologists in their diagnostic tasks.

As demonstrated by Wu et al. [26] CTTA could be used to rapidly differentiate COVID-19 from other infectious pneumonia. However, radiomic signature has been proven effective in classifying between stable and progressive group of COVID-19 patients with an AUC, sensitivity and specificity of 0.8, 81, and 74%, respectively [25]. Our study highlights the feasibility of CTTA in the COVID-19 patient management, with a performance higher than the mere visual assessment. CTTA is readily applicable as an adjunct tool in a routine clinical setting whether the preliminary results from our pilot study could be confirmed on larger scale. Our results may potentially reflect histopathological differences in parenchymal findings; the different inflammatory infiltrate induced by the SARS-CoV could explain the differences on CTTA in patients with COVID-19 disease compared to those with same CT findings related to the other interstitial pneumonia, as demonstrated by the lower CT density in positive COVID-19 subjects.

The implementation of CTTA might strengthen the role of Chest CT during COVID-19 pandemic, potentially improving patient management in the emergency department [45].

Despite the encouraging results, the study has limitations related to its retrospective nature, its single-center design, the small sample size and the lack of inter-reader agreement due to in consensus reading. Nevertheless, previous studies have demonstrated good reproducibility for filtration-histogram-based CTTA using multicenter clinical validation [35, 46] and robustness to variation in image acquisition parameters [47, 48]. It is worth noting that the contour of the GGO was drawn on a single axial slice. Although the multi-slice or volume delineation of GGO would be a better representation within the whole lung, such methodology is time-consuming and therefore not practical in clinical setting. Furthermore, the comparable results in heterogeneity assessment have been reported between cross-sectional area and whole volume analysis on CT [49]. Lastly, the absence of histopathologic correlation did not allow for confirmation of the above hypothesis between the significant CTTA parameters and the tissue/parenchymal alterations caused by SARS-CoV-2 and immune system response.

In conclusion, CTTA parameters with and without filtration such as SD, MPP, and kurtosis can help to differentiate between positive and negative COVID-19 patients with higher specificity than simple visual assessment and could potentially be used as an adjunct tool during radiological interpretation of Chest CT.

## Data Availability

The datasets during and/or analyzed during the current study available from the corresponding author on reasonable request.

## Abbreviations

AUC: Area under the curve
COVID-19: Coronavirus Disease-19
CT: Computed tomography
CTTA: CT texture analysis
DICOM: Digital imaging and communications in medicine
GGO: Ground-glass opacity
HU: Hounsfield unit
IRB: Institutional review board
LoG: Laplacian of Gaussian
MPP: Mean of positive pixels
PACS: Picture archiving and communication system
ROC: Receiver operating characteristic
ROI: Region of interest
RT-PCR: Real-time reverse transcription polymerase chain reaction
SARS-CoV-2: Severe Acute Respiratory Syndrome Coronavirus 2
SD: Standard deviation
SSF: Spatial scale of the filter

## Code availability

Texture analysis was performed by using a dedicated software (TexRAD, Feedback Medical Ltd., Cambridge, UK).
